# Vaccinations versus Lockdowns to Prevent COVID-19 Mortality

**DOI:** 10.3390/vaccines10081347

**Published:** 2022-08-18

**Authors:** Ronen Arbel, Joseph Pliskin

**Affiliations:** 1 Maximizing Health Outcomes Research Lab, Sapir College, Sderot 7956000, Israel; 2 Community Medical Services Division, Clalit Health Services, Tel-Aviv 6209804, Israel; 3 Departments of Health Policy and Management, Industrial Engineering and Management, Ben-Gurion University of the Negev, Beer-Sheva 84105, Israel; 4 Department of Health Policy and Management, Harvard T. H. Chan School of Public Health, Boston, MA 432-2217, USA

**Keywords:** COVID-19 vaccinations, mortality, lockdown

## Abstract

Measures employed to combat COVID-19 included public lockdowns and vaccination campaigns. Israel’s extensive public health system produced data demonstrating the real-world results of these measures. Our objective was to evaluate the health and economic outcomes of the measures to cope with COVID-19. Publicly available datasets from the Israeli Ministry of Health were used to model the parameters of the pandemic in Israel. The Oxford COVID-19 Government Response Tracker was used for quantitative data on government policies. Data on the Israeli economy were taken from the Central Bureau of Statistics. Our models demonstrate that the first lockdown prevented 1022 COVID-19 deaths at the cost of 36.4–38.6 billion NIS. The second lockdown prevented 1970 COVID-19 deaths and cost 18–21 billion NIS. These lifesaving effects were observed with a time lag from the declaration of lockdown. The primary vaccination campaign cost 1 billion NIS and prevented 4750 COVID-19 deaths. The first vaccination booster campaign prevented 650 COVID-19 deaths and cost 51.1 million NIS. Therefore, the cost per prevented COVID-19 death is 10–36 million NIS with a national lockdown versus 210,000 NIS in the primary vaccination campaign and 79,000 NIS in the first booster campaign. In conclusion, both lockdowns and vaccination campaigns effectively lower COVID-19 deaths, but the cost to avoid one COVID-19 death with effective vaccination is 50–466 times lower than with a lockdown.

## 1. Introduction

Coronavirus disease 2019 (COVID-19) is caused by the novel coronavirus (SARS-CoV-2). As of June 2022, over 500 million cases and 6 million deaths have been confirmed [1]. Non-pharmaceutical interventions (NPIs) such as social distancing are crucial in reducing SARS-CoV-2 infections [2]. Prior to the development of treatment or vaccine, NPIs effectively contained the disease’s spread. Countries that experienced recent infectious disease outbreaks, such as China, Taiwan, The Republic of Korea, and Singapore, were more prepared than Western countries [3]. Lockdowns are an NPI measure, but not the only one; testing and contact tracing is an NPI measure applied in the Asian countries mentioned, which has helped limit spread. However, the most used NPI was lockdown. In 2020, Israel posed three nationwide lockdowns that effectively reduced COVID-19 mortality [4]. However, in early 2021, with the development of effective mRNA vaccines, Israel adopted a strategy of ultra-fast deployment of vaccinations, especially for the older adult population. Israel was also the first to deploy the first and second booster vaccination campaigns. This strategy proved to be highly effective in preventing COVID-19 mortality and avoided additional lockdowns. Evidence about the economic implications of utilizing a lockdown strategy versus a massive mRNA vaccination strategy for preventing death due to COVID-19 is limited. Therefore, our objective was to compare the cost needed to prevent one case of COVID-19 mortality by lockdowns and vaccinations.

## 2. Materials and Methods

### 2.1. Study Population

The entire Israeli population (about 9 million residents).

### 2.2. Outcomes

The study’s primary outcome was the cost of preventing one COVID-19-related mortality event.

### 2.3. Strategies to Prevent Death Due to COVID-19

The measures to cope with COVID-19 in Israel that were analyzed in the study are as follows:

1. Nationwide lockdowns, as defined by an Oxford Stringency Index of 80% or higher [5]. The lockdown included school closure, workplace closure, cancelation of public events, restriction on gatherings, closure of public transport, requirements to stay at home, restriction on internal movement and traveling, and international traveling control.
2. Mass vaccination campaigns, defined as efforts to vaccinate the general adult population as quickly as possible.

### 2.4. Study Perspective

The design was from the perspective of the Israeli government.

### 2.5. The Time Horizon for Economic Evaluation

The time horizon for the economic evaluation was usually held at three months due to the apparent impact of lockdown on mortality.

### 2.6. Discount Rate

Due to the short time horizon (3 months), cost and benefit estimates were not discounted.

### 2.7. Data Sources, Collection, and Encoding Method

For the study, several dedicated databases were constructed. The clinical and economic data from the sources detailed in Table 1 were integrated into these databases. The variables analyzed are only those obtained from a reliable and publicly available data source.

### 2.8. Data Organization

All data were extracted, analyzed, cleaned, and organized into several unique databases we made public. The data processing code is publicly available on our Github site (https://github.com/maxcorlabs/Corona_Cost_Effectiveness, accessed on 15 August 2022).

The following datasets were published publicly on Zenodo: COVID-19 vaccination data in Israel by age over time until August 2021 [10]. Four tabular datasets, derived from publicly available Israel Ministry of Health (MOH) data but processed for analytics about uptake in different age groups over time, are included in the following DOI [11]. They cover the primary mass vaccination campaign for COVID-19 until August 2021. Three of the datasets are of a specific ordinal vaccination, i.e., datasets of only first, second, or third doses.A dataset created from processing various official Israel MOH datasets and the Oxford Stringency Index for Israel by MaxCor labs from the start of the pandemic until 1 April 2021, is included in this DOI. Hospital data on patients, testing result data, vaccination data, and OSI are all in one dataset.

### 2.9. Measurement of Effectiveness

The primary outcome of this research is the health benefits of implementing differing health policies in Israel. This outcome was primarily measured by the total number of deaths avoided due to the implementation of health policies.

### 2.10. Prediction Models for the Effect of Lockdowns

We approximate lives saved by lockdowns by the formula:

$$\overset{M=\left(y+90\right)}{\underset{M=y}{\sum }}\left(Dm+C\right)-\overset{N=\left(x+90\right)}{\underset{N=x}{\sum }}\left(Dn+C\prime \right)$$

where *Dm* denotes daily deaths without a lockdown, summed from the first day of the period without lockdown (*y*) until *y* + 90, *Dn* denotes daily deaths with a lockdown, summed for each day *N* from the first day of lockdown (*x*) until *x* + 90, *C* denotes the mortality rate when entering the lockdown, calculated as the mean number of daily deaths in the first 30 days of the lockdown, and *C*′ is the adjustment of *C* to mortality rates outside of Israel.

We developed several prediction models to calculate the number of deaths avoided from the measures implemented in Israel. Our simplest models were based on various naïve time series models, and other simpler models included curve fitting and polynomial regressions. All models used real-world observational data as inputs.

### 2.11. Measurement of Costs—Lockdowns

The cost of implementing the measures was defined as the loss in GDP in New Israeli Shekels (NIS) for the policy’s period.

To monetize the primary outcome, we divided the loss in GDP by the total number of mortality cases prevented, resulting in the cost per mortality case prevented.

### 2.12. Measurement of Effectiveness—Vaccination Campaigns

We used published studies that evaluated the effectiveness of preventing COVID-19 mortality in the primary vaccination [12] in Israel and the first booster campaigns in Israel’s largest HMO [13].

### 2.13. Measurement of Costs—Vaccination Campaigns

As the economy of Israel was practically open during the primary and booster vaccination campaigns [6], we calculated the direct costs of vaccinations during the relevant periods. The calculation for the booster campaign was based on published estimates [14].

## 3. Results

Estimation of the effectiveness of lockdown is detailed in the following DOI [15]. It includes three separate datasets built on data from processing various official Israel MOH datasets and the Oxford Stringency Index for Israel, along with a coded notebook of the calculations and documentation explaining the calculations. The hospital data on patients, testing results data, vaccination data, and OSI are included and summed for daily, weekly, and monthly datasets.

A summary of the results of estimated costs, prevented COVID-19 deaths, and estimated cost per prevented COVID-19 death in the two lockdowns and 2 vaccination campaigns are summarized in Table 2.

## 4. Discussion

### 4.1. Summary of Results

Our models demonstrate that the first lockdown prevented 1022 COVID-19 deaths at the cost of 36.4–38.6 billion NIS (equivalent to 10.5–11.2 billion USD). The second lockdown prevented 1970 COVID-19 deaths and cost 18–21 billion NIS (equivalent to 5.2–6.1 billion USD). These lifesaving effects were observed with a time lag from the declaration of lockdown. The primary vaccination campaign cost 1 billion NIS (equivalent to USD 0.29 billion) and prevented 4750 COVID-19 deaths. The first vaccination booster campaign prevented 650 COVID-19 deaths and cost 51.1 million NIS. Therefore, the cost per prevented COVID-19 death is 10–36 million NIS (equivalent to 3.1 to 10.6 million USD) with a national lockdown versus 210,000 NIS (USD 60,846) in the primary vaccination campaign and 79,000 NIS (USD 22,744) in the first booster campaign.

### 4.2. Comparison of Methods and Results to the Literature

The subject of measuring the means implemented to cope with COVID-19 is still under development. Greenstone and Nigam suggested [16] calculating the benefits of preventing mortality cases. They first estimated the number of mortality cases prevented and then monetized the prevented cases. The estimated number of mortality cases was based on the Ferguson study [17] that used two scenarios. The first scenario included a mitigation scenario that combines home isolation of suspected cases, home isolation for individuals living in the same house as the suspected cases, and social distancing of elderly and individuals at high risk; the second scenario was a no-policy scenario. The prevented cases were then divided into nine age groups and then monetized using the United States (US) Government’s Value of a Statistical Life—VSL (individuals’ willingness to pay to reduce the risk of death) adjusted for age. Following this approach, the authors found that 1.7 million mortality cases could have been avoided in the US mitigation strategy. The authors have monetized this clinical benefit to an economic benefit to the extent of 8 trillion US dollars. This approach’s main limitation was that the economic burden associated with these mitigation strategies was overlooked, specifically, their effect on the labor market, private consumption, and GDP.

Broek-Altenburg and Atherly [18] evaluated the benefits of such measures in terms of quality-adjusted life-years (QALYs). Firstly, the authors used the median age of death caused by COVID-19 in Italy at 80.5 and used American citizens’ life expectancy at that age. Secondly, they estimated the cost of implementing a moderate social distancing policy as the federal incentive package’s value in the US valued at 1 trillion US dollars. According to their calculations, the value per QALY in the US was estimated to be between USD 75,000 and USD 650,000.

Kim and Neumann [19] compared those two approaches and pointed out the importance of assessing all the benefits and costs in implementing the means to cope with COVID-19. These include health outcomes, healthcare costs, labor market, consumption, legal/crime justice, education, and environment. The authors emphasized that research in this area should detail which costs and benefits were considered and excluded from the analyses.

Thunström et al. [20] compared the benefits and costs of ‘flattening the curve’ in the US through social distancing policies defined as the avoidance of gathering of 10 or more people and travel restrictions that resulted in a temporary closure of schools, universities, and public events. They defined benefits as the number of lives saved due to social distancing. They monetized them using the average willingness to pay for a reduction in mortality risk. The costs were defined as the difference in GDP loss’ present value with and without social distancing. The outcome was the benefit–cost ratio between the value of lives saved and the total GDP loss. They reported a net benefit of USD 5.16 trillion from social distancing. The study had several significant limitations, including using a single policy package, ignoring the distributional impact of such measures on different groups within the society, and the probability of a second wave due to preventing herd immunity through social distancing.

Miles et al. [21] conducted a cost–benefit analysis of the UK lockdown and compared the results with European countries with similar healthcare resources and income. The lockdown’s economic impact was measured as a percentage loss in GDP and the benefits as a gain in life expectancy adjusted for poor health and quality of life. To monetize the lockdown’s health benefits, they applied a value of GBP 30,000 per QALY, the maximum NICE threshold. The significant finding of this cost–benefit analysis was that no matter how many lives were saved, even at the high estimate of 400,000 lives saved, the cost always exceeded the benefits. This cost–benefit analysis concluded that the lockdown policy implemented in the UK was not beneficial since the costs exceeded the benefits and recommended targeted restrictions on high-risk groups instead of the whole population.

### 4.3. Limitations

Our study has several limitations. The primary limitation is that calculating the precise number of prevented COVID-19 deaths by lockdowns and vaccines is challenging. In case the measures were not implemented, the estimation of potential events is subject to numerous potential biases, both for vaccinations and lockdowns and especially for combinations of the two measures. For example, we assumed and observed that the government removed most lockdown restrictions due to the high uptake of the COVID-19 vaccine in Israel. Moreover, a new wave of COVID-19 mortality due to lower uptake of vaccinations may force the government to return to the lockdown strategy so that we may overestimate mortality without any vaccinations. Despite the potential bias of our estimates during the primary vaccination campaign compared to no vaccination, they were lower than predicted deaths from the results of simple curve-fitting to model the situation with no vaccines and no lockdowns.

Other reasons for bias may be various sociodemographic and clinical confounders related to the vaccine uptake and effectiveness, Therefore, our estimation of the first booster campaign was based on detailed patient-level data from Clalit Health Services (CHS), considered one of the most reliable sources of COVID-19 vaccination effectiveness worldwide [11]. CHS and Israeli data have been the primary source of evidence for policy-makers worldwide due to the fast roll-out of the primary vaccination [22] and the first and second booster campaigns [23].

### 4.4. Policy Implications

To create the best possible public health outcomes for Israel and worldwide, policymakers must carefully weigh a health policy’s financial effects [24]. Measures against former epidemics in Israel have not led to such negative financial consequences as the initial emergency measures against COVID-19, which involved lockdowns of public life. The results of such financial consequences will be seen for decades to come in every area of life, including public health.

Although the primary mass vaccination campaign was successful, as the Delta variant appeared, deaths began to rise despite high vaccination rates. The scientific debate about the extent that this rise was due to vaccinations waning and to what extent the Delta variant was less preventable with the current vaccination regime continues, The debate was settled with growing evidence of the effectiveness of the boosters during the Delta wave [13], which overcame the vaccines’ waning immunity. Policymakers worldwide faced a similar challenge with the emergence of the Omicron wave in late 2021. Again, a waning immunity of the first booster was evident, and a second booster, initiated again in Israel, demonstrated a significant reduction in COVID-19 mortality [23].

In the midst of 2022, the world is again facing a new wave of COVID-19—this time as a result of the Omicron BA.4 and BA.5 strains [25]. If we can learn from the past, we expect that a third booster will be needed to cope with this wave.

Based on our measures of the economic impacts of various strategies, we recommend that actions that exclude public lockdowns should be used against the epidemic as it unfolds, including education campaigns, increased research, and most importantly, mass vaccination campaigns before public lockdowns are considered.

## 5. Conclusions

The cost per prevented COVID-19 death is USD 3,100,000–USD 10,600,000 with a national lockdown versus USD 22,744 to USD 63,000 with massive vaccination: a 47–466-fold difference between the strategies.

The most effective and economical strategy to prevent COVID-19 mortality is maintaining maximal vaccination protection of the high-risk population, using mRNA vaccines and boosters.

## Figures and Tables

**Table 1:** Clinical and economic data included in the analyses.

	Variables	Description	Source
Clinical variables	Daily mortality due to COVID-19, age, sector, gender, death date		Ministry of Health [6]
Policy-related variable	Overall Stringency Index value (0–100%)	Uses 17 indicators categorized into four categories: Containment and closure policies Economic policies Health system policies Miscellaneous policies Values range from 0–100%	Oxford University [5]
	Number of daily vaccinations		Ministry of Health [6]
Economic variables	Quarterly value of GDP for the years 2020, 2021	GDP values and economic forecast	Central Bureau of Statistics, Ministry of Finance, Central Bank, Israel [7,8,9]

**Table 2:** Comparison of the cost needed to prevent one COVID-19 mortality.

Period	COVID-19 Measure	Estimated COVID-19 Deaths Avoided	Estimated Cost (NIS)	Estimated Cost to Avoid one COVID-19 Death (NIS)
April–June 2020	First Israeli lockdown	1022	37,500,000,000	36,692,759
September–November 2020	Second Israeli lockdown	1970	21,000,000,000	10,659,898
March–June 2021	Primary vaccination campaign	4750	1,000,000,000	210,526
August–September 2021	First booster campaign	650	51,150,221	78,693

## Data Availability

Links to all the data and analysis in the research were provided and referenced in the manuscript.
